# The role of the catecholic and the electrophilic moieties of caffeic acid in Nrf2/Keap1 pathway activation in ovarian carcinoma cell lines

**DOI:** 10.1016/j.redox.2014.11.012

**Published:** 2014-12-05

**Authors:** R. Sirota, D. Gibson, R. Kohen

**Affiliations:** Faculty of Medicine, Institute for Drug Research, Hebrew University of Jerusalem, Israel

**Keywords:** CA, caffeic acid, HCA, dihydrocaffeic acid, DMCA, dimethylcaffeic acid, Nrf2, nuclear factor E2-related factor 2, Keap1, Kelch ECH associating protein 1, EpRE, electrophile response element, GSH, glutathione, GST, glutathione-S-transferase, GSR, glutathione reductase, HO-1, heme oxygenase 1, NQO1, NAD(P)H quinone oxidoreductase 1, Coffee, Caffeic acid, Nrf2, Cisplatin, Polyphenols

## Abstract

In recent years, numerous studies have demonstrated the health benefits of polyphenols. A major portion of polyphenols in western diet are derived from coffee, which is one of the most consumed beverages in the world.

It has been shown that many polyphenols gain their beneficial properties (e.g. cancer prevention) through the activation of the Nrf2/Keap1 pathway as well as their direct antioxidant activity. However, activation of Nrf2 in cancer cells might lead to resistance towards therapy through induction of phase II enzymes.

In the present work we hypothesize that caffeic acid (CA), a coffee polyphenol, might act as an electrophile in addition to its nucleophilic properties and is capable of inducing the Nrf2/EpRE pathway in cancer cells.

The results indicate that CA induces Nrf2 translocation into the nucleus and consequently its transcription. It has been demonstrated that generated hydrogen peroxide is involved in the induction process. It has also been found that this process is induced predominantly *via* the double bond in CA (Michael acceptor). However, surprisingly the presence of both nucleophilic and electrophilic moieties in CA resulted in a synergetic activation of Nrf2 and phase II enzymes.

We also found that CA possesses a dual activity, although inducing GSTP1 and GSR, it inhibiting their enzymatic activity.

In conclusion, the mechanism of induction of Nrf2 pathway and phase II enzymes by CA has been elucidated. The electrophilic moiety in CA is essential for the oxidation of the Keap1 protein. It should be noted that while the nucleophilic moiety (the catechol/quinone moiety) can provide scavenging ability, it cannot contribute directly to Nrf2 induction. It was found that this process may be induced by H_2_O_2_ produced by the catechol group.

On the whole, it appears that CA might play a major role in the cancer cells by enhancing their resistance to treatment.

## Highlights

• The electrophilic moiety in CA is essential for the oxidation of the Keap1 protein.
• The nucleophilic moiety contribute to Nrf2/Keap1 activation via production of H_2_O_2_.
• CA possesses a dual activity, as inducer and as inhibitor of GSTP1 and GSR1.
• The effect of coffee on healthy subjects and cancer patients may be different.

## Graphical abstract

**Figure:**
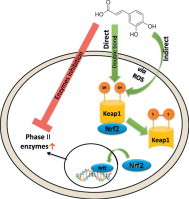

## Introduction

Coffee is one of the most popular and consumed beverages in the world. A solid epidemiological data show that daily coffee consumption is associated with a decreasing risk for many types of cancers [1–4]. The risk for type II diabetes is also decreased by daily coffee consumption [5]. These properties may be attributed to high polyphenol content of coffee as it considered a major source of polyphenol intake in the western diet.

Polyphenols are a group of phytochemicals that are found in many foods. Today about 8000 compounds are identified, most of them natural antioxidants [6,7]. Besides their antioxidant activity, many polyphenols have other beneficial health properties such as antibacterial [8,9], anti inflammatory [10,11] and cancer preventive [12,13]. We showed in a previous work that coffee polyphenols act as antioxidants in the GI tract [14].

Coffee is rich with polyphenols originating from the hydrocinnamic family, mainly chlorogenic and caffeic acids [15]. The best known component of coffee, caffeine, constitutes only 1–2.5% of dry coffee beans, while polyphenols can reach 10% and more of coffee dry weight [16]. Most of the chlorogenic acids are metabolized in the colon to free caffeic or ferulic acids [17].

Many polyphenols, such as curcumin, epigallocatechin gallate and resveratrol, although differing in structure, gain their beneficial properties through the activation of the Nrf2/Keap1 pathway [18–20].

Under normal conditions, Nrf2 is targeted by Keap1, which promotes Nrf2 proteasomal degradation *via* interactions with ubiquitin ligase. Keap1 further functions as a sensor of stress signals, through stress-induced oxidation of key cysteine residues that lead to conformational changes which promotes Nrf2 release from Nrf2/Keap1 complex and translocation of the former to the nucleus. In the nucleus Nrf2 interacts with basic leucine zipper transcription factors such as Maf and Jun family members and binds to a cis-acting element, the electrophile response element (EpRE or antioxidant response element-ARE) [21]. Nrf2–EpRE is a major pathway which regulate phase II antioxidant responses, stress responses and cell proliferation. EpRE inducers consist of several types of chemicals, including (1) redox-active bisphenols, quinones, and phenylenediamines; (2) Michael acceptors; (3) isothiocyanates; (4) dimercaptans; (5) hydroperoxides; (6) metals; (7) polyphenols and more [22].

Nrf2 is found to induce phase II cytoprotective genes related to cellular stress response, such as the heme oxygenase 1 (HO-1), ferritin, NAD(P)H quinone oxidoreductase (*NQO1*), thioredoxin and glutathione metabolizing genes such as: glutathione s-transferases (GSTs), γ-glutamyl transpeptidase (*GGT*), *GSR*, *GPx* and glutamyl cysteine ligase (GCL) [23].

Nrf2 activation is considered nowadays as a double edged sword. On one hand, Nrf2 plays an essential role in maintaining cellular homeostasis and hence represents a critical target for prevention of oxidative stress or inflammation associated carcinogenesis [24]. On the other hand, over expression or higher ratio of nuclear/cytosolic Nrf2 may give advantage to cancer cells in coping with various cytotoxic drugs via the expression of phase II detoxifying enzymes [25]. For example, tumors over expressing *GSTP1* are found to be in high correlation with tumor resistance towards cisplatin treatment [26].

The GSTs are a multigene family of isoenzymes involved in the detoxification of endogenous as well as exogenous substances such as chemotherapeutic drugs by conjugation of GSH to the electrophilic site of the substance [27]. *GST* has been shown to be overexpressed in various human cancer tissues made resistant to chemotherapeutic drugs [28,29]. Hence, activation of Nrf2 in cancer cells might lead to resistance towards radiation and chemotherapy through induction of phase II enzymes. Coffee, therefore, may be cancer preventive but it might reduce the efficiency of chemotherapy through phase II gene induction during treatment of the ongoing disease.

**Fig. 1:**
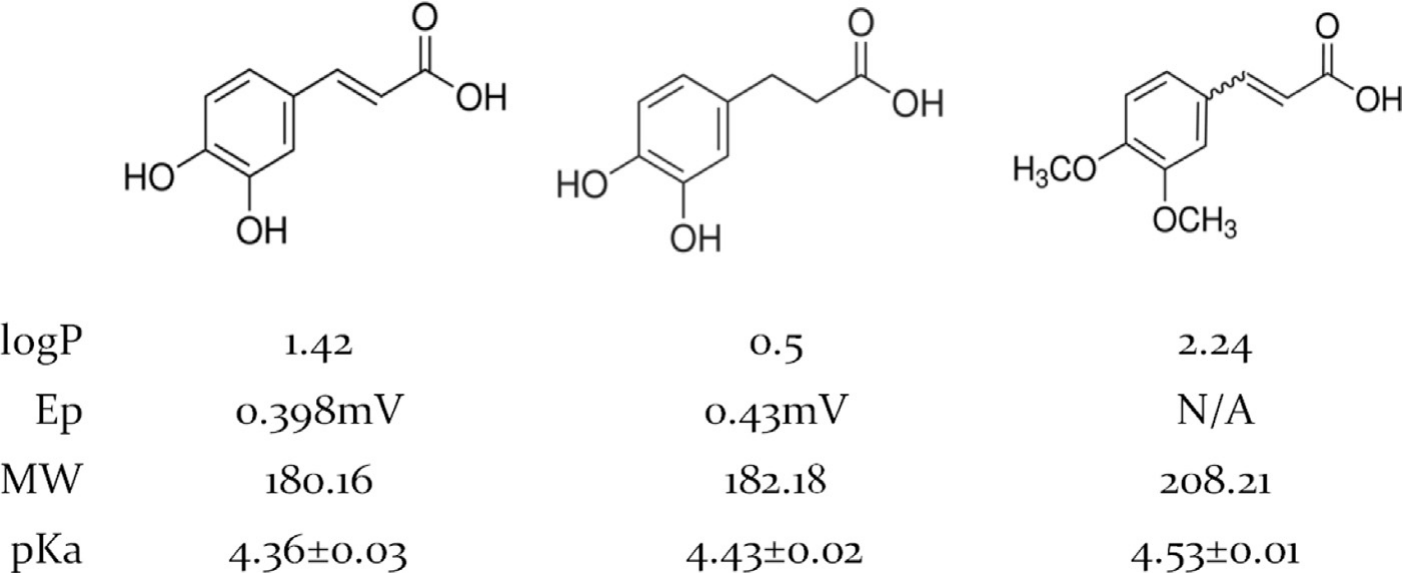
Structures and chemical properties of CA and its derivatives, HCA and DMCA (left to right).

Caffeic acid (CA) possesses both nucleophilic (catechol) moiety and electrophilic (Michael acceptor) moiety. It is believed that polyphenols induce Nrf2 not in their nucleophilic form, but rather in the oxidized form (quinone) after electron/s donation. H_2_O_2_ is generated during the oxidation of polyphenols in aqueous media in the presence of oxygen [30]. H_2_O_2_ is a known inducer of Nrf2, and thus it is possible that polyphenols induce Nrf2 only, indirectly, via H_2_O_2_ [31]. In order to understand which group of CA is responsible for Nrf2 induction we chose two CA derivatives: dihydrocaffeic acid (HCA) which consists of the polyphenolic group only and dimethylcaffeic acid (DMCA) consisting of only the Michael acceptor group (Fig. 1). In the present work we hypothesize that caffeic acid (CA), might act as an electrophile in addition to its nucleophilic properties and is capable of inducing the Nrf2/EpRE pathway in cancer cells. We also set to reveal the differences of phase II gene induction and activities in different cells, thus we chose 2 cell lines: ovarian carcinoma cells, A2780 and cisplatin resistant daughter cell line, A2780cisR. These findings may provide us with further understanding of the structure–activity relation of Nrf2 induction by polyphenols and the beneficial or harming properties of coffee consumption during cancer treatment.

## Materials and methods

### Materials

Caffeic acid (CA), 3,4-dihydrocinnamic acid (HCA), 3,4-dimethoxycinnamic acid (DMCA), 1-chloro-2,4-dinitrobenzene (CDNB), reduced glutathione (GSH), oxidized glutathione (GSSG), gly–gly, l-γ-glutamyl-p-nitronilide, NADPH, ATP, cysteine, glutamine, serine, 2,3 naphthalene dicarboxaldehyde (NDA), 5-sulfosalicylic acid (SSA), 3-(4,5-dimethylthiazole-2-yl)-2,5-diphenyl tetrazolium bromide (MTT), enzymes and other common reagents were purchased from Sigma Chemical Co., St. Louis, MO. Primary and secondary antibodies were purchased from Abcam, Cambridge, MA. RNA isolation kits, cDNA kits and qRT-PCR reagents were purchased from Agentek, Israel.

### Cell culture

Human ovarian carcinoma cells A2780 and the daughter line A2780cisR were obtained from ATCC, USA. The cells were cultured in RPMI 1640 medium supplemented with 10% fetal bovine serum, 2 mM l-glutamine, and 50 µg/ml gentamycin. The cultures were maintained in a humidified 5% CO_2_ incubator at 37 °C. Cells were subcultured every 3–4 days to maintain logarithmic growth and were allowed to grow for 24 h before use.

### RT-PCR

2×10^5^ Cells were seeded per well in 6 wells dish to examine the effect of the test compounds on the transcription of Nrf2 and other phase II genes. All compounds were dissolved in PBS. Following incubation, the culture medium was removed and the cells were rapidly rinsed with ice-cold PBS. Total RNA was extracted with TRI Reagent (Sigma). 1 Microgram of RNA was reverse transcribed at 37 °C for 120 min using primers as described below using Omniscript Reverse Transcription Kit (QIAGEN). cDNA obtained from the reverse transcriptase (RT) reaction (amount corresponding to 1 µg of total RNA) was subjected to PCR using QuantiTect SYBR Green PCR (QIAGEN). PCR reaction parameters were as follows: heating to 50 °C for 2 min, incubation at 95 °C for 10 min and thereafter 40 cycles of denaturation at 94 °C for 15 s, annealing at 60 °C for 60 s and extension at 72 °C for 30 s. This relative quantification compares the PCR signal of the target transcript to the endogenous control gene GAPDH. Primers: Nrf2 forward 5′-ATT TCT CCC AAT TCA GCC AGC CCA-3′ Nrf2 reverse 5′-TAC AAA CGG GAA TGT CTG CGC CAA-3′ HO-1 forward 5′-GGC AGA GAA TGC TGA GTT CAT GAG GA-3′ HO-1 reverse 5′-ATA GAT GTG GTA CAG GGA GGC CAT CA-3′ GAPDH forward 5′-TCG ACA GTC AGC CGC ATC TTC TTT-3′ GAPDH reverse 5′-ACC AAA TCC GTT GAC TCC GAC CTT-3′ GSTP1 forward 5′-GGC TAG GAC CTC ATG GAT CA-3′ GSTP1 reverse 5′-ACC TCC GCT GCA AAT ACA TC -3′ NQO1 forward 5′-AAG CCG CAG ACC TTG TGA TAT TCC-3′ NQO1 reverse 5′-AAC ACT CGC TCA AAC CAG CCT TTC-3′ GCLM forward 5′-ACA GCT GTT GAC TCA CAA TGA TCC-3′ GCLM reverse 5′-GTG CGC TTG AAT GTC AGG AAT GCT-3′ GSR1 forward 5′-GCC GCC TGA ATG CCA TCT ATC AAA-3′ GSR1 reverse 5′-TAT TGT GGG CTT GGG ATC ACT CGT-3′.

### Immunochemical staining

Cells were seeded on sterilized cover slips 24 h prior to treatments. Following treatments the cover slips were washed 4 times with 0.05% Tween in PBS and fixed with 4% formaldehyde in PBS for 10 min and washed twice with cold PBS. For permeabilization, cells were incubated in 0.25% Triton X-100 for 10 min. Cells were blocked by 10% goat serum in PBST containing 0.3 M glycine for 30 min.

Cells were then incubated overnight at 4 °C with primary rabbit-antihuman Nrf2 antibody (Abcam, Cambridge, Massachusetts) diluted according to manufacturer's instructions. Cells were washed with PBS 3×5 min and incubated with secondary FITC-goat antirabbit antibody (Abcam, Cambridge, Massachusetts, USA) for 1 h at RT in the dark, washed and counterstained with 0.5 µg/ml Hoechst33258 (Abcam, Cambridge, Massachusetts, USA) for 10 min in the dark. The slips were mounted and read on FluoView FV10i confocal microscope (Olympus, Hamburg, Germany). The nucleus/cytosol ratio was calculated using ImageJ software by dividing FITC intensity in the nuclei area by FITC intensity in the cytosol area of at least 5 cells in each slip, 6 slips for each treatment.

### Western blotting

Protein expression in A2780 and A2780cisR cells was analyzed as described previously [32]. The proteins were extracted in denaturation buffer (Tris pH 6.8, 20% SDS, glycerol and mercaptoethanol) and separated on 13% acrylamide gel. The primary antibodies were purchased as follow: GCLM, GSR, GST3/GSTP, β-tub (Rabbit monoclonal Abcam, Cambridge, Massachusetts, USA). β Tubulin was used as loading control.

Primary antibodies were detected using a horseradish peroxidase-conjugated antibody and enhanced chemiluminescence (Abcam, Cambridge, Massachusetts, USA). The films were scanned, and band intensity was quantified using densitometry software (ImageJ, National Institutes of Health, Bethesda, Maryland, USA).

### Enzyme activity assays

The cells were washed following the treatments with ice-cold PBS and then homogenized with 0.5 ml cold homogenization buffer (50 mM potassium phosphate pH 7.4, 1.15% KCl). The homogenates were frozen overnight at 80 °C, sonicated (3 cycles of 10 s with 5 s intervals) and centrifuged at 10,000*g* for 20 min at 4 °C. Protein content was measured by the method of Bradford et al. [33] using bovine serum albumin as a standard.

GST activity, expressed as % of control, was determined according to Habig et al. [34]. The assay was performed using potassium phosphate buffer (pH 6.5) with reduced glutathione (GSH) and 1-chloro-2,4-dinitrobenzene. Activity was calculated from the changes in absorbance at 340 nm (*ε*340 nm=9.6 mM^−1^ cm^−1^).

GSR activity was determined according to the method described by Beutler et al. [35]. The assay was performed using 0.5 mM EDTA, 0.10 M sodium phosphate buffer (pH 7.6) with oxidized GSH and NADPH. Activity was calculated from the changes in absorbance at 340 nm (*ε*340 nm=6.22 mM^−1^cm^−1^).

GCL activity was determined according to the method described by White et al. [36]. GCL reaction cocktail (400 mM Tris, 40 mM ATP, 20 mM l-glutamic acid, 2.0 mM EDTA, 20 mM sodium borate, 2 mM serine, 40 mM MgCl_2_) were pipetted into wells. The GCL reaction was initiated by adding cysteine (dissolved in TES/SB) to each GCL activity well (cysteine was not added to the GSH-baseline wells at this time). The plate was then vortexed, covered, and incubated for 30 min. The GCL reaction was terminated by adding SSA to all wells, and then 2 mM cysteine was added to the GSH-baseline wells. The plate was then vortexed and held on ice for 20 min. Following protein precipitation, the plate was centrifuged for 5 min at 2500 rpm on an Eppendorf centrifuge. Following centrifugation, aliquots of supernatant from each well of the reaction plate were transferred to a 96-well plate designed for fluorescence detection. Next, NDA derivatization solution (50 mM Tris, pH 10, 0.5 N NaOH, and 10 mM NDA in DMSO, v/v/v 1.4/0.2/0.2) was added to all wells of this plate. The plate was covered to protect the wells from light and allowed to incubate at room temperature for 30 min. Following incubation, fluorescence intensity was measured (472 ex/528 em) on a fluorescence plate reader (Bio-Tek synergy HT plate reader).

### DCF assay

Cells were seeded in 96 well plates in 100% confluence. In darkness, 100 µM of H_2_DCF in fresh cell medium added for 30 min at 37 °C. The cells were washed twice with PBS then treated for 30 min (including tBHP standards for calibration). The cells were washed again 3 times and 0.1 ml of DMSO:PBS (9:1) was added and the plates were shaken at RT for 10 min. Plates were measured (exs. 485–490/em 520–530) on a fluorescence plate reader (Bio-Tek synergy HT plate reader). The results presented as % of control (normalized arbitrary fluorescence units).

### Anoxic experiments

Cells were seeded on 6 well plates for 24 h prior to the experiment in a humidified 5% CO_2_ incubator at 37 °C. Then the cells were treated and transferred to an ischemia chamber as previously described by Portugal-Cohen et al. [37]. Briefly, the chamber was saturated with 95% N_2_–5% CO_2_, oxygen levels were monitored the entire time (0.7–1 mg/l) and the system was heated to 37 °C by hot water circulation. Incubation period was 6 h.

### Statistical analysis

The results are expressed as means±S.E. and were analyzed using repeated measurements of one-way ANOVA followed by Dunnett’s *t*-test. *P* values less than 0.05 were considered statistically significant. All analyses were conducted using Stat version 3.01 (Graph Pad Inc.).

## Results

### Nrf2 translocation

In order to find out whether CA can imitate the Nrf2/Keap1 pathway, we first examined its ability to induce translocation of the Nrf2 protein into the nucleus. It is known that the ratio of nucleic/cytosolic Nrf2 is important for the phase II genes transcription.

**Fig. 2:**
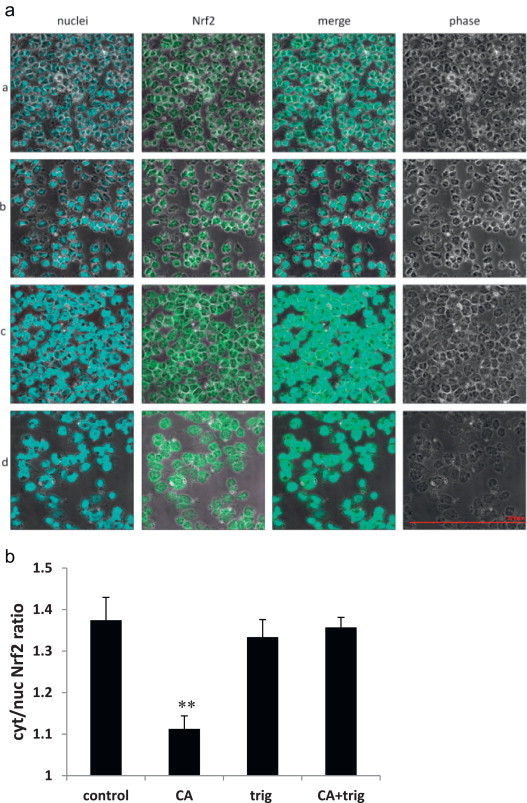
Effect of CA on Nrf2 translocation in A2780 cells. The figure is a representative (*n* = 6) result for confocal laser-scanning. (a) A2780 cells stained for Nrf2 (FITC-green) and for nuclei (Hoescht-blue). The cells were either untreated (panel A) or incubated with 50 µM CA (panel B), 1 µM trigonelline (panel C) or both (panel D) for 6 h at 37 °C in an atmosphere of 5% (v/v) CO_2_. (b) Ratio between cytoplasmic and nuclear Nrf2 measured by color analysis (Fluoview1 software, Olympus, Hamburg, Germany). *p*=0.003 CA vs. control. All pictures are in the same scale.

A2780 cells were measured in fluorescence microscope following their staining with FITC fluorescent dye for Nrf2 and Hoechst 33258 dye for the nuclei (Fig. 2a). Fig. 2b shows that in the control cells Nrf2 is located both in the nucleus and in the cytosol with cyt/nuc ratio of 1.37. Following treatment with 50 µM of CA, Nrf2 is translocated into the nucleus and the ratio is significantly reduced to 1.11. Trigonelline, an alkaloid known for its inhibitory effect on Nrf2 translocation [38] prevented the translocation of NRf2 induced by CA. It can be seen that at 1 µM trigonelline, most of the Nrf2 remained in the cytosol at a ratio of 1.33. Combination of CA and trigonelline showed results similar to the control.

### Nrf2 and phase II genes expression in A2780/cisR cells following CA, HCA, and DMCA treatment

**Fig. 3:**
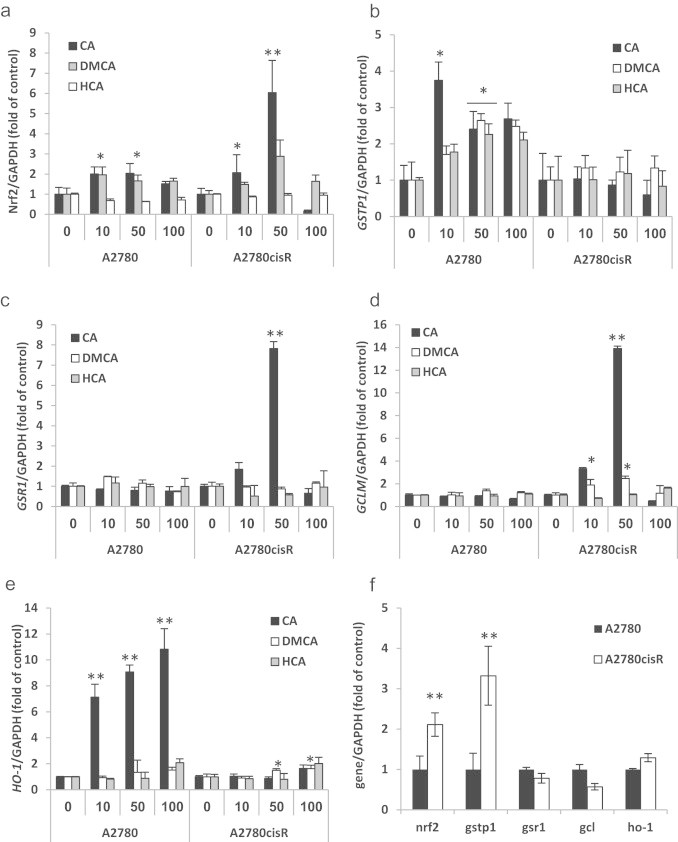
Effects of CA (black), DMCA (white) and HCA (gray) treatments on Nrf2 and phase II mRNA levels in A2780/cisR cells. (a) Effects of CA, DMCA and HCA treatments on Nrf2 mRNA levels; (b) effects of CA, DMCA and HCA treatments on *GSTP1* mRNA levels; (c) effects of CA, DMCA and HCA treatments on *GSR1* mRNA levels; (d) effects of CA, DMCA and HCA treatments on *GCLM* mRNA levels; (e) effects of CA, DMCA and HCA treatments on *HO-1* mRNA levels; (f) basal mRNA levels of untreated cells. Data are expressed as *n*-fold of control (untreated cells) normalized to GAPDH ±SD of 6 independent experiments each conducted in triplicate, ^⁎^*p*<0.05; ^⁎⁎^*p*<0.005.

Nrf2, GSH metabolizing enzymes and other phase II gene expressions were assessed by qRT-PCR. In A2780 cells, Nrf2 mRNA level was induced to a 2-fold expression following treatment with CA and DMCA. However, as shown in Fig. 3a, HCA was unable to induce expression of the Nrf2 mRNA. In the A2780cisR cells, the basal level of Nrf2 was twice as high as the sensitive cells (Fig. 3f). It can also be seen that those cells are much more susceptible for induction by CA. The Nrf2 mRNA levels were 2.1 and 6.0 fold higher than the control when treated with 10 and 50 µM CA, respectively. DMCA treatment resulted in a milder effect of 1.5 and 2.9 fold expression compared to the control at 10 and 50 µM, respectively. Again, HCA had no effect on Nrf2 mRNA levels (Fig. 3a).

*GSTP1* mRNA levels were ~4 fold higher than the control after 10 µM of CA treatment in A2780 cells. Higher concentrations induced only 2.5 fold overexpression. DMCA and HCA showed dose response expression with maximum expression of 2.5 fold of the control (Fig. 3b). In the resistant cells (Fig. 3f) the basal level was 3 fold higher than A2780 cells control and all the compounds, regardless of their concentration, did not induce *GSTP1* mRNA expression levels nor down regulate them (Fig. 3b).

*GSR1* mRNA levels showed no significant changes in the sensitive cells following treatments with the three compounds. However, in the resistant cells, CA induced 1.8 and 7.8-fold overexpression at 10 and 50 µM, respectively. No other compound induced any change in *GSR1* expression (Fig. 3c). Notably, at high concentration (100 µM), the mRNA levels of most genes tested were as the basal levels or even lower indicating a bell-shaped behavior.

No changes in *GCLM* expression were observed in the sensitive cell line following the various treatments. CA treatment of 10 and 50 µM resulted in a 3.3 and 13.9-fold expression of *GCLM* mRNA in the resistant cells (Fig. 3d). DMCA at same concentrations resulted in 1.9 and 2.5-fold expression, and HCA had no effect on *GCLM* mRNA levels.

*HO-1* demonstrated the most significant enhancement in mRNA induction following the various treatments. As shown in Fig. 3e, treatment with CA in different concentrations 10, 50 and 100 µM resulted in 7.1, 9.1 and 10.8-fold expression mRNA. However, in the resistant cells, CA induced *HO-1* only by 1.6 fold of the control at 100 µM. DMCA, which did not demonstrate any induction in the sensitive cells, induced *HO-1* by 1.5 and 1.6 fold at 50 and 100 µm, respectively. A significant, 2.0-fold expression was observed for 100 µM of HCA.

### Effect of Nrf2 import inhibitor, trigonelline, on CA action

**Fig. 4:**
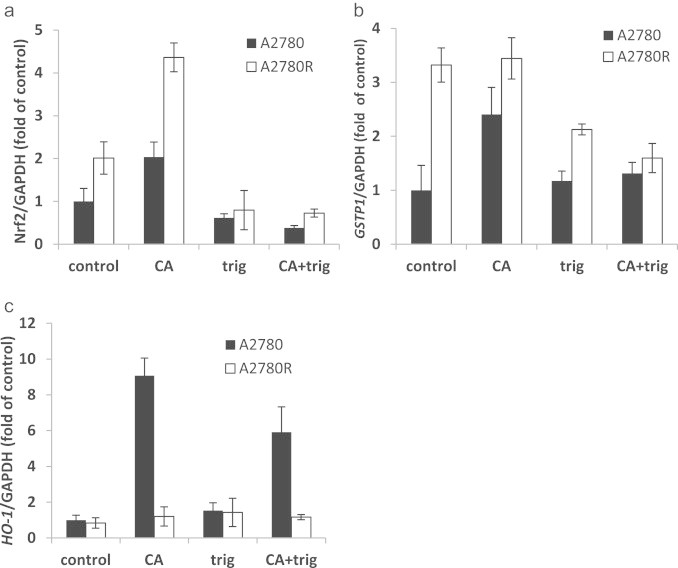
Inhibition of Nrf2 and phase II genes induction by trigonelline in A2780 and A2780cisR cells. Cells were treated with 50 µM CA, 1 µM trig or in combination of both for 6 h and mRNA levels were measured by qRT-PCR. (a) Nrf2 mRNA levels after CA and/or trigonelline treatments for 6 h in A2780 cells (black) and in A2780cisR cells (white); (b) *GSTP1* mRNA levels after CA and/or trigonelline treatments for 6 h in A2780 cells (black) and in A2780cisR cells (white); (c) *HO-1* mRNA levels after CA and/or trigonelline treatments for 6 h in A2780 cells (black) and in A2780cisR cells (white). *n*=3; data represented as mean±SD. ^⁎^*p*<0.05.

To validate the results from the Nrf2 translocation experiment, we measured the mRNA levels of Nrf2, *GSTP1* and *HO-1* at the same conditions as mentioned above with and without trigonelline. CA increased Nrf2 level by 2 and 4.5 fold in A2780 and A2780cisR, respectively. Trigonelline alone had no effect on Nrf2 levels in A2780 cell but reduced Nrf2 by 2 fold in A2780cisR cells (resistant cells). The combination of both, the same as in trigonelline treatment, completely abolished the effect of CA (Fig. 4a). Trigonelline demonstrated similar effects on *GSTP1* mRNA levels. In A2780 (sensitive cells) it had no effect on its own but in combination with CA *GSTP1* mRNA remained at the basal level. In A2780cisR (resistant) trigonelline reduced the relatively high basal levels of GSTP1 mRNA levels by 36%. Similar results were obtained when CA and trigonelline were administrated together (Fig. 4b).

*HO-1* mRNA levels were not affected in A2780cisR cells. However, in the sensitive cells *HO-1* mRNA levels were highly induced by CA (up to 9 fold of the control). Co-administration of CA with trigonelline diminished the induction by 34% (Fig. 4c).

### Phase II protein levels following CA treatment

Western blot analysis of GST3/GSTP in A2780 cells showed a trend of dose–response behavior following CA treatment, although not statistically significant. CA increased GST levels by 40% and 60% at 10 and 100 µM, respectively. Similar to the findings of mRNA levels, the protein levels of GST in A2780cisR cells were higher (25–30%) than in A2780 cells. In those cells no further induction was evident.

CA had no effect on GSR levels in A2780 cells, but showed a bell-shaped response in the resistant cells with a maximum increase of 20% at 50 µM.

GCLM was elevated by 15% and 30% at 10 and 100 µM, respectively, in A2780 cells. Again in the resistant cells the levels were higher in untreated cells (by 25%) and were unchanged following CA treatments.

It is noteworthy that GST and GSR protein translation were in correlation with their mRNA levels following CA treatments. However, such correlation was not observed for GCLM protein.

### Phase II enzymes activities following CA treatment

In order to induce phase II enzymes and evaluate their activity, the cells were treated with CA, the most potent Nrf2/Keap1 pathway inducer. The activities were normalized to the control (no treatment) of the two cell lines.

It has been found that GSTP1 activity in the sensitive cells demonstrated a bell-shaped curve. The maximum enzyme activity (250% vs. control) was observed at 50 µM concentration of CA. At 100 µM (Fig. 6a), the activity significantly decreased towards the base line. Surprisingly, when the resistant cells were treated with CA, an opposite bell shape was observed. At 50 µM CA the activity of the enzyme decreased significantly (~50% of control) and returned to baseline at 100 µM treatment (Fig. 6b).

The activity of GSR1 following treatment with various concentrations of CA was decreased to 40–50% of the control in the sensitive cells (A2780, Fig. 6a). No effect on GSR1 activity was detected in the resistant cells (Fig. 6b).

The activity of GCL in the sensitive line was increased slightly by 20% at 10 and 50 µM of CA treatment and further by 50% at treatment of 100 µM (Fig. 6a). A moderate increase (40%) was observed in the resistant cells only following treatment of cells with CA at 100 µM (Fig. 6b).

In vitro activity assays of the three enzymes show that CA inhibits the activity of GST and GSR with IC_50_ of 95 and 70 µM, respectively (Fig. 6c).

### Cellular oxidative stress following CA, HCA and DMCA

Polyphenols undergo oxidation to form semiquinones or quinones and subsequently produce H_2_O_2_. As Nrf2/Keap1 system possesses oxidative stress sensor activities, it is possible that the observed Nrf2 activation might be due to the production of reactive oxygen species (ROS) (e.g. hydrogen peroxide) and not due to the polyphenol *per se*. ROS formation was measured in the treated cells (following 6 h of incubation) by H_2_DCFDA/DCF method [39]. The results in Table 1 show that none of the 3 compounds increased the ROS levels compared to the control. It was even suggested that ROS levels were reduced by 13% at 100 µM of the various compounds although the changes are not statistically significant. tBHP, a positive control, at 50 µM elevated ROS levels by 3 fold of the control.

### Nrf2 and phase II genes expression in H_2_O_2_ and O_2_ free environments

While no evidence for increasing ROS levels was detected, the involvement of low efflux of hydrogen peroxides and other ROS produced by CA in low concentrations in cell culture media cannot be disregarded as shown previously by Halliwell [40].

To explore the role of ROS generated by CA outside the cells (CA barely penetrated the cells, but did as other polyphenols and bound to the outer membrane [41,42]) and its effect on Nrf2 pathway induction, we conducted two experiments.

1. Catalase (300U) was added to cell media and 2. The cells were treated under anoxic conditions.

The addition of catalase to A2780 cell media had no effect on the activation of the Nrf2 pathway by DMCA. However, it reduced the induction of the Nrf2 induced by CA by 30% (Fig. 7a). Similar results were observed for *GSTP1*and *HO-1* (Figs. 7b and 6c respectively).

Nrf2 mRNA levels remained unchanged following 6 h incubation of the cells with DMCA in an anoxic chamber. However, the ability of CA to induce Nrf2 was reduced in anoxic environments compared to normoxia conditions.

The results were similar to the results obtained in the catalase experiments (sensitive cells) (Fig. 8). In these cells *GSTP1* was induced under anoxic condition by 3.5 fold, CA treatment under these conditions had no further effect on *GSTP1* levels.

## Discussion

Coffee has been found to be cancer preventive for many cancer types. This phenomenon was attributed to the antioxidant scavenging properties of coffee polyphenols but can be also attributed to Nrf2 induction by these compounds. Nrf2/Keap1 is a hormetic pathway, in which low doses of inducers may lead to susceptibility for ROS accumulation and oxidative stress. However, sustained or high dosage of inducers might lead to drug resistance and give advantage to cancerous cells in their microenvironment. However, a transient induction by polyphenols (or other inducers) will lead to an increase in phase II enzymes and therefore may prevent diseases including cancer [43]. It is now established that Nrf2 and inducible phase II genes play an important role in cancer development, progression and sensitivity to chemotherapeutic treatments.

We found that caffeic acid, a coffee polyphenol, is capable of inducing the Nrf2 pathway in A2780and A2780cisR cells. We suggested that although caffeic acid possesses reducing properties (due to its phenolic group) it can act as an electrophile as well. The double bond in its structure (Fig. 1) might act as a Michael acceptor and attract electrons. Moreover, caffeic acid can be oxidized to the quinone form, which can act also as an electrophile directly or indirectly. These electrophilic properties may lead to the activation of the Nrf2/Keap1 pathway due to oxidation of the thiol groups on the Keap1 protein. These findings encouraged us to explore the mechanism by which CA induces Nrf2 and subsequently phase II enzymes.

In order to determine the specific moieties responsible for the induction of phase II enzymes a structure–activity relationship of caffeic acid was conducted. It was also of interest to determine whether CA can evoke such cancer protection mechanisms in cancer cells sensitive to chemotherapeutic compounds and cells which are resistant to such treatment. Therefore, 2 cell lines were chosen: A2780 cell (sensitive to cisplatin treatment) and A2780cisR cells (resistant to treatment). We also speculated that caffeic acid which barely penetrates the cells, may act indirectly by producing hydrogen peroxide outside the cells which in turn penetrates and evokes the Nrf2/Keap1 response.

Immunocytochemical staining of Nrf2 in A2780 cell showed that CA led to translocation of Nrf2 to the nucleus (Fig. 2). This effect was completely abolished by co-treatment with trigonelline, an inhibitor of Nrf2 translocation [38]. Trigonelline also inhibited the mRNA expression of Nrf2, *GSTP1* and *HO-1* confirming that those genes are under the control of Nrf2 pathway.

In order to explore which part of CA is responsible for the initiation of the Nrf2 pathway and the translocation of Nrf2 into the nucleus, three different molecules were studied; CA (double bond and phenolic groups), DMCA (without the phenolic groups), and HCA (without the double bond). It can be concluded that the Michael acceptor moiety (the double bond) is necessary for Nrf2/Keap1 and subsequently phase II gene induction (Fig. 3). Catechol group alone possess reducing properties and therefore, it was not expected that this compound would initiate the Nrf2 pathway. Surprisingly, it has been found that the combination of both groups (phenolic and double bond) acted synergistically to activate the Nrf2 response (CA vs. DMCA).

Nrf2 mRNA basal levels found to be twice as high in the resistant cells than in the sensitive ones, in agreement with Bao et al. [44]. In these cells Nrf2 was also much more inducible, especially by CA, with an increase of 6 fold. This finding may suggest that resistant cells are intrinsically more capable of dealing with stressors. The strong induction of Nrf2 and subsequently the protecting phase II enzymes may provide these cells with the ability, at least partly, to overcome cisplatin cytotoxicity. Lister et al. demonstrated that Nfr2 is 10-fold overexpressed in pancreatic cancer cells than in normal pancreatic ductal cells [45]. Here we compared Nrf2 levels from two different lines of cancer cells. It can be speculated that the differences in Nrf2 levels between cisplatin resistant cells and healthy ovarian cells may be even more pronounced.

It has been suggested that one of the mechanisms of cellular resistance towards cisplatin is detoxification of the drug by GSH adduction which could be facilitated by the GSTs enzymes. Even though cisplatin–GSH adducts found in *in vitro* experiments and were separated from whole cells, it has also been found that GSTPs are very slow catalysts of these reactions [46]. The authors concluded that GST is too slow to produce cisplatin–GSH adducts in living cells. Nevertheless, a correlation was found between GST levels and susceptibility to cisplatin resistance in cancer patients [47].

In the present work we showed that there is a 3-fold increase in the basal levels of *GSTP1* in the resistant line *vs.* the sensitive cells. It should be noted that the pattern of induction of the gene between the 2 cell lines is different. While there was no further induction in the resistant cells, in the sensitive cells, CA and DMCA induced *GSTP1* to the same level as the basal level of resistant cells. Same pattern was observed at the protein level.

It seems that *GSTP1* reaches a certain saturation which limits its further induction and may be high enough to provide protection. Recently, GSTs were linked to two noncatalytic pathways critical to cell survival. GST involved in S-glutathionylation of key proteins, including self S-glutathionylation, is an inhibitor of JNK signaling pathway [48]. It can be speculated that a tight regulation on GST levels is preserved because GSTs enzymes are a multi-functional family controlling not only cell detoxification but also key survival factors.

GSR1 recycles oxidized GSH in the cells. GSR1 basal mRNA and protein levels in both cell lines were found to be equal. The sensitive cells were unresponsive to induction at all concentrations of CA unlike the resistant cells in which 50 µM CA induced a 6-fold overexpression. Hirano et al., found that overexpression of riboflavin kinase which is involved in the first step in the FAD synthesis (FAD is an essential co enzyme for GSR) renders cell resistance not only to cisplatin but also to hydrogen peroxide [49]. Cisplatin induces oxidative stress and hydrogen peroxide production in cells and is believed to be one of the mechanisms underlying cisplatin-induced nephrotoxicity. The results suggest that the sensitive cells are not as adaptive to oxidative stress as the resistant cells and thus are more susceptible to hydrogen peroxide induced apoptosis (data not shown).

A different pattern of RNA induction by CA was observed for *HO-1*. In contrast to the previous results indicating significant induction of the various phase II enzymes' mRNA in the resistant cells, only a 2-fold increase was observed for *HO-1* at high concentrations of CA and DMCA as shown in Fig. 3e. However, when the sensitive cells were treated with CA, a 10-fold increase in *HO-1* mRNA levels in a dose dependent manner was recorded. HO-1 facilitates the catabolism of heme to CO, ferrous iron and biliverdin which are consequently reduced by biliverdin reductase to bilirubin, each of which acts via distinct molecular targets to influence cell function as proliferation, apoptosis and coping with oxidative stress. Many studies have shown that HO-1 induction attenuates cisplatin-induced nephrotoxicity [50] and ototoxicity *via* the reduction of oxidative stress [51,52]. The results obtained for *HO-1* are paradoxical since *HO-1* was highly overexpressed in cisplatin sensitive cells following CA treatment but not in resistant cells. Thus, induced overexpression of *HO-1* might be positive in the reduction of cisplatin side effects but not necessarily lead to resistance towards the drug.

Although we demonstrated the necessity of the double bond (Michael acceptor) in CA molecule to induce the Nrf2/Keap1 pathway, the mechanism by which the phenolic groups contribute to this induction (see previous section, the combination of both groups, phenolic and double bond, acted synergistically to activate the Nrf2 response) was elucidated. Therefore, the possibility that CA can produce ROS in the cell interior (H_2_O_2_ for example) which in turn can activate the Nrf2/Keap1 pathway was studied.

The results obtained from the DCF experiments did not indicate an enhanced production of ROS inside the cells by the three CA derivatives studied. However, using this methodology may not be sensitive enough to detect minor changes in ROS levels. Therefore, the possibility that CA bound to a cell's outer membrane can undergo autoxidation and produce hydrogen peroxide in small quantities which in turn can diffuse into the cell and evoke the Nrf2/Keap1 response was investigated. Therefore, catalase, which cannot penetrate into the cells, was added to remove hydrogen peroxide in cell surroundings. The result showed attenuation of Nrf2 induction by CA by 40% indicating the involvement of exogenous hydrogen peroxide. The fact that DMCA, which does not contain phenolic groups and cannot undergo autoxidation, was not affected by the presence of catalase indicates that DMCA can only activate the pathway *via* the double bond and supports the assumption that CA can activate the Nrf2/Keap1 pathway by these two mechanisms. Therefore, generated H_2_O_2_ plays an important role in CA-mediated Nrf2 activation.

To further explore the importance of ROS produced extracellularly or intracellularly in activation of the Nrf2/Keap1 pathway, the cells were treated with CA under anoxic conditions. The results show clearly that CA can induce Nrf2/Keap1 pathway in the sensitive cells under these conditions indicating that the double bond (Michael acceptor) alone is sufficient to induce a response and also suggests that CA, at least partially, can penetrate the cell. The effect of CA and DMCA at low oxygen conditions was similar to that observed when a catalase was used in the aerobic conditions.

**Fig. 5:**
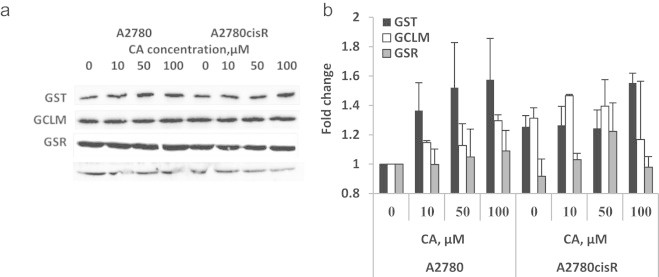
Glutathione-S-transferase (GST), glutathione reductase (GSR) and glutamate–cysteine ligase-modifier subunit(GCLM) protein levels in A2780 and A2780cisR cell following CA treatment for 16 h representative western blots of GST, GCLM and GSR (a) and quantification (b). β Tubulin was used as loading control. Results expressed as fold of control (sensitive cells) of 3 independent experiments.

**Fig. 6:**
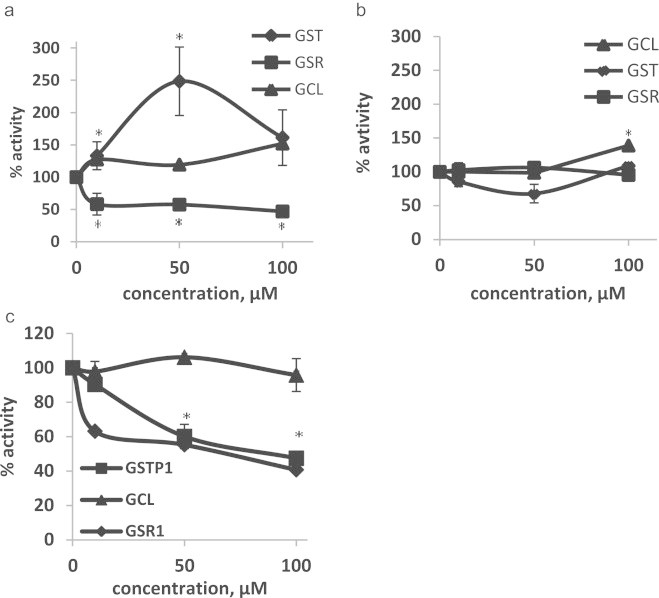
Glutathione-S-transferase (GST), glutathione reductase-1 (GSR) and glutamate–cysteine ligase (GCL) activities in A2780 and A2780cisR cell following CA treatment. (a) Enzymes activities in A2780 cells. (b) Enzymes activities in A2780cisR cells and (c) *in vitro* enzymes activities. Data are expressed as % of control±SD of 3 independent experiments each conducted in triplicate. ^⁎^*p*<0.05 comparing untreated cells.

**Fig. 7:**
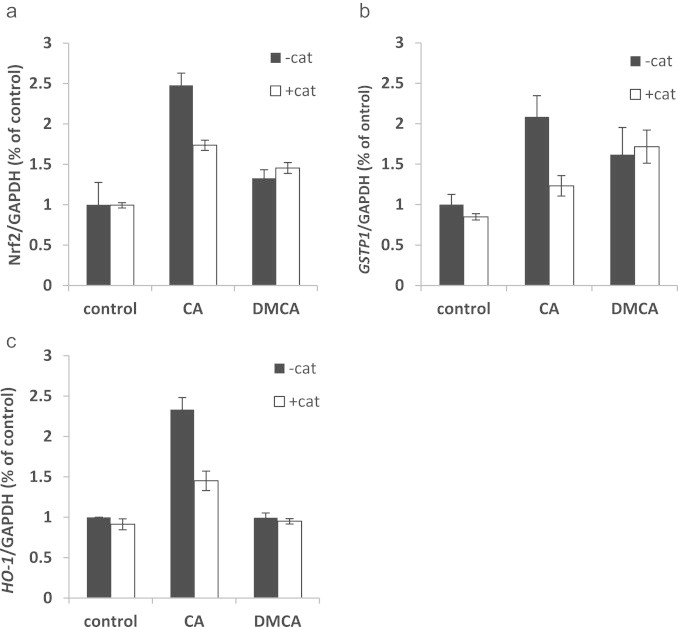
Phase II genes expression in A2780 cells after CA and DMCA treatment in the presence of catalase. (a) Nrf2 mRNA levels after CA and DMCA 6 h treatments (black) and the same treatments in presence of 300 U of catalase in cell media (white); (b) *GSTP1* mRNA levels after CA and DMCA 6 h treatments (black) and the same treatments in presence of 300 U of catalase in cell media (white); (c) *HO-1* mRNA levels after CA and DMCA 6 h treatments (black) and the same treatments in presence of 300 U of catalase in cell media (white); *n*=3; data represented as mean±SD.

**Fig. 8:**
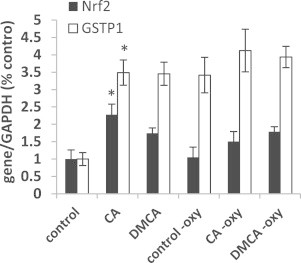
Nrf2 and GSTP1 expression in A2780 cells after CA and DMCA treatments under normal growth condition and in the absence of oxygen. Nrf2 (black) and *GSTP1* (white) mRNA levels after CA and DMCA treatments for 6 h. *n*=4; data represented as mean±SD. ^⁎^*p*<0.05 comparing untreated cells.

As can be seen, although most genes are up-regulated by CA and sequentially the proteins levels increased (Fig. 5), the enzyme activity of some of the enzymes in the cells decreased comparing to the control. The most dramatic effect was observed in the GSTP1 activity in A2780 (sensitive cells) compared to A2780cisR cells (resistant cells). A possible explanation for the bell shaped activity of GST in A2780 cells may be the competition between induction and inhibition. Indeed, we found that CA inhibits GST and GSR activity *in vitro*. This finding is in agreement with a previous work of Ploemen et al. [53]. GSR1 activity in A2780 cells (in these cells no increase in protein was observed) was inhibited by 50% but not in the resistant cells in which the protein levels were increased. Thus, CA possesses a dual mechanism of action, it can act as a *GSTP1* and *GSR1* inducer and also as their inhibitor. This finding may indicate that CA may be used as a potential chemotherapeutic adjuvant due to its ability to rescue healthy cells from drug induced oxidative stress and also, in the right concentration, prevent the activity of GST and therefore, prevent the inactivation of chemotherapeutics drugs.

In conclusion, the mechanism of induction of Nrf2 and phase II enzymes by CA has been elucidated. The electrophilic moiety in CA is essential for the oxidation of the Keap1 protein inside the cell. Although, the nucleophilic moiety (the catechol/quinone) can provide scavenging ability, it can also contribute to Nrf2/Keap1 activation via production of hydrogen peroxide intracellularly or extracellularly.

In the cancer cells which are resistant to cisplatin, the basal levels of some of the phase II enzymes are high and different from control both in the gene and in the protein levels. In these cells CA possesses a different pattern of induction. Finally, CA possesses a dual activity, as initiator of the Nrf2/Keap1 pathway and as inhibitor of GSTP1.

Therefore, the effect of coffee on healthy subjects and cancer patients may be different. The use of coffee in people under chemotherapeutic treatment should be carefully examined since it contains both Nrf2 inducers and an inhibitor.

## Conflict of interest

The authors have no conflicts of interest.

## Figures and Tables

**Table 1:** ROS levels in A2780/cisR cells after CA, HCA and DMCA treatments normalized to control.

	**A2780**				**A2780cisR**		
	**10** **µM**	**50** **µM**	**100** **µM**		**10** **µM**	50 **µM**	**100** **µM**
**CA**	94.1±9.5	93.4±4.0	89.0±4.0		97.4±12.2	94.0±4.2	88.3±2.4
**DMCA**	94.1±8.0	94.6±5.3	91.6±3.3		94.3±4.8	97.3±5.7	92.0±6.1
**HCA**	89.4±2.9	88.2±2.6	88.0±3.0		89.4±4.2	90.7±1.3	86.7±4.4
**tBHP**^a^		312.1±7.0			301.2±9.1		

a tBHP=tert-butylhydroperoxide.

## References

[1] Radoï L., Paget-Bailly S., Menvielle G., Cyr D., Schmaus A., Carton M. (2013). Tea and coffee consumption and risk of oral cavity cancer: results of a large population-based case-control study, the ICARE study. Cancer Epidemiology.

[2] Sang L.X., Chang B., Li X.H., Jiang M. (2013). Consumption of coffee associated with reduced risk of liver cancer: a meta-analysis. BMC Gastroenterology.

[3] Tian C., Wang W., Hong Z., Zhang X. (2013). Coffee consumption and risk of colorectal cancer: a dose–response analysis of observational studies. Cancer Causes Control.

[4] Wilson K.M., Bälter K., Möller E., Adami H.O., Andrén O., Andersson S.O. (2013). Coffee and risk of prostate cancer incidence and mortality in the cancer of the prostate in Sweden Study. Cancer Causes & Control.

[5] Higdon J.V., Frei B. (2006). Coffee and health: a review of recent human research. Critical Reviews in Food Science and Nutrition.

[6] Pannala A.S., Rice-Evans C.A., Halliwell B., Singh S. (1997). Inhibition of peroxynitrite-mediated tyrosine nitration by catechin polyphenols. Biochemical and Biophysical Research Communications.

[7] Rice-Evans C.A., Miller N.J. (1996). Antioxidant activities of flavonoids as bioactive components of food. Biochemical Society Transactions.

[8] Brown J.C., Jiang X. (2013). Activities of muscadine grape skin and polyphenolic constituents against *Helicobacter pylori*. Journal of Applied Microbiology.

[9] Rashed K.N., Butnariu M. (2014). Isolation and antimicrobial and antioxidant evaluation of bio-active compounds from *Eriobotrya japonica* stems. Advanced Pharmaceutical Bulletin.

[10] Chao C.Y., Mong M.C., Chan K.C., Yin M.C. (2010). Anti-glycative and anti-inflammatory effects of caffeic acid and ellagic acid in kidney of diabetic mice. Molecular Nutrition & Food Research.

[11] Hwang S.J., Kim Y.W., Park Y., Lee H.J., Kim K.W. (2014). Anti-inflammatory effects of chlorogenic acid in lipopolysaccharide-stimulated RAW 264.7 cells. Inflammation Research.

[12] Darvesh A.S., Bishayee A. (2013). Chemopreventive and therapeutic potential of tea polyphenols in hepatocellular cancer. Nutrition and Cancer.

[13] Malerba S., Galeone C., Pelucchi C., Turati F., Hashibe M., La Vecchia C. (2013). A meta-analysis of coffee and tea consumption and the risk of glioma in adults. Cancer Causes & Control.

[14] Sirota R., Gorelik S., Harris R., Kohen R., Kanner J. (2013). Coffee polyphenols protect human plasma from postprandial carbonyl modifications. Molecular Nutrition & Food Research.

[15] Erk T., Hauser J., Williamson G., Renouf M., Steiling H., Dionisi F. (2014). Structure- and dose-absorption relationships of coffee polyphenols. Biofactors.

[16] Manach C., Scalbert A., Morand C., Rémésy C., Jiménez L. (2004). Polyphenols: food sources and bioavailability. American Journal of Clinical Nutrition.

[17] Vitaglione P., Fogliano V., Pellegrini N. (2012). Coffee, colon function and colorectal cancer. Food & Function.

[18] Han S.G., Han S.S., Toborek M., Hennig B. (2012). EGCG protects endothelial cells against PCB 126-induced inflammation through inhibition of AhR and induction of Nrf2-regulated genes. Toxicology and Applied Pharmacology.

[19] Hao E., Lang F., Chen Y., Zhang H., Cong X., Shen X. (2013). Resveratrol alleviates endotoxin-induced myocardial toxicity via the Nrf2 transcription factor. PLOS One.

[20] Zhao R., Yang B., Wang L., Xue P., Deng B., Zhang G. (2013). Curcumin protects human keratinocytes against inorganic arsenite-induced acute cytotoxicity through an NRF2-dependent mechanism. Oxidative Medicine and Cellular Longevity.

[21] Zhang D.D. (2006). Mechanistic studies of the Nrf2–Keap1 signaling pathway. Drug Metabolism Reviews.

[22] Bryan H.K., Olayanju A., Goldring C.E., Park B.K. (2013). The Nrf2 cell defence pathway: Keap1-dependent and -independent mechanisms of regulation. Biochemical Pharmacology.

[23] Zhang M., An C., Gao Y., Leak R.K., Chen J., Zhang F. (2013). Emerging roles of Nrf2 and phase II antioxidant enzymes in neuroprotection. Progress in Neurobiology.

[24] Acharya A., Das I., Chandhok D., Saha T. (2010). Redox regulation in cancer: a double-edged sword with therapeutic potential. Oxidative Medicine and Cellular Longevity.

[25] Wang X.J., Sun Z., Villeneuve N.F., Zhang S., Zhao F., Li Y. (2008). Nrf2 enhances resistance of cancer cells to chemotherapeutic drugs, the dark side of Nrf2. Carcinogenesis.

[26] Pasello M., Michelacci F., Scionti I., Hattinger C.M., Zuntini M., Caccuri A.M. (2008). Overcoming glutathione S-transferase P1-related cisplatin resistance in osteosarcoma. Cancer Research.

[27] Dourado D.F., Fernandes P.A., Ramos M.J. (2010). Glutathione transferase classes alpha, pi, and mu: GSH activation mechanism. Journal of Physical Chemistry B.

[28] Lu D., Shi H.C., Wang Z.X., Gu X.W., Zeng Y.J. (2011). Multidrug resistance-associated biomarkers PGP, GST-pi, Topo-II and LRP as prognostic factors in primary ovarian carcinoma. British Journal of Biomedical Science.

[29] Schumaker L., Nikitakis N., Goloubeva O., Tan M., Taylor R., Cullen K.J. (2008). Elevated expression of glutathione S-transferase pi and p53 confers poor prognosis in head and neck cancer patients treated with chemoradiotherapy but not radiotherapy alone. Clinical Cancer Research.

[30] Halliwell B. (2008). Are polyphenols antioxidants or pro-oxidants? What do we learn from cell culture and in vivo studies?. Archives of Biochemistry and Biophysics.

[31] Pecorelli A., Bocci V., Acquaviva A., Belmonte G., Gardi C., Virgili F. (2013). NRF2 activation is involved in ozonated human serum upregulation of HO-1 in endothelial cells. Toxicology and Applied Pharmacology.

[32] Soroka Y., Sagi A., Khalaila I., Abdu U., Milner Y. (2000). Changes in protein kinase C during vitellogenesis in the crayfish *Cherax quadricarinatus* − possible activation by methyl farnesoate. General and Comparative Endocrinology.

[33] Bradford M.M. (1976). A rapid and sensitive method for the quantitation of microgram quantities of protein utilizing the principle of protein–dye binding. Analytical Biochemistry.

[34] Habig W.H., Pabst M.J., Jakoby W.B. (1974). Glutathione S-transferases. The first enzymatic step in mercapturic acid formation. Journal of Biological Chemistry.

[35] Beutler E., Duron O., Kelly B.M. (1963). Improved method for the determination of blood glutathione. Journal of Laboratory and Clinical Medicine.

[36] White C.C., Viernes H., Krejsa C.M., Botta D., Kavanagh T.J. (2003). Fluorescence-based microtiter plate assay for glutamate–cysteine ligase activity. Analytical Biochemistry.

[37] Portugal-Cohen M., Kohen R. (2009). Exposure of human keratinocytes to ischemia, hyperglycemia and their combination induces oxidative stress via the enzymes inducible nitric oxide synthase and xanthine oxidase. Journal of Dermatological Science.

[38] Arlt A., Sebens S., Krebs S., Geismann C., Grossmann M., Kruse M.L. (2013). Inhibition of the Nrf2 transcription factor by the alkaloid trigonelline renders pancreatic cancer cells more susceptible to apoptosis through decreased proteasomal gene expression and proteasome activity. Oncogene.

[39] Wang G., Gong Y., Burczynski F.J., Hasinoff B.B. (2008). Cell lysis with dimethyl sulphoxide produces stable homogeneous solutions in the dichlorofluorescein oxidative stress assay. Free Radical Research.

[40] Halliwell B. (2014). Cell culture, oxidative stress, and antioxidants: avoiding pitfalls. Biomedical Journal.

[41] Fadel O., El Kirat K., Morandat S. (2011). The natural antioxidant rosmarinic acid spontaneously penetrates membranes to inhibit lipid peroxidation in situ. Biochimica et Biophysica Acta.

[42] Sato Y., Itagaki S., Kurokawa T., Ogura J., Kobayashi M., Hirano T. (2011). In vitro and in vivo antioxidant properties of chlorogenic acid and caffeic acid. International Journal of Pharmaceutics.

[43] Calabrese V., Cornelius C., Dinkova-Kostova A.T., Calabrese E.J., Mattson M.P. (2010). Cellular stress responses, the hormesis paradigm, and vitagenes: novel targets for therapeutic intervention in neurodegenerative disorders. Antioxidants & Redox Signaling.

[44] Bao L.J., Jaramillo M.C., Zhang Z.B., Zheng Y.X., Yao M., Zhang D.D. (2014). Nrf2 induces cisplatin resistance through activation of autophagy in ovarian carcinoma. International Journal of Clinical and Experimental Pathology.

[45] Lister A., Nedjadi T., Kitteringham N.R., Campbell F., Costello E., Lloyd B. (2011). Nrf2 is overexpressed in pancreatic cancer: implications for cell proliferation and therapy. Molecular Cancer.

[46] Peklak-Scott C., Smitherman P.K., Townsend A.J., Morrow C.S. (2008). Role of glutathione S-transferase P1-1 in the cellular detoxification of cisplatin. Molecular Cancer Therapeutics.

[47] Rolland D., Raharijaona M., Barbarat A., Houlgatte R., Thieblemont C. (2010). Inhibition of GST-pi nuclear transfer increases mantle cell lymphoma sensitivity to cisplatin, cytarabine, gemcitabine, bortezomib and doxorubicin. Anticancer Research.

[48] Tew K.D., Townsend D.M. (2011). Regulatory functions of glutathione S-transferase P1-1 unrelated to detoxification. Drug Metabolism Reviews.

[49] Hirano G., Izumi H., Yasuniwa Y., Shimajiri S., Ke-Yong W., Sasagiri Y. (2011). Involvement of riboflavin kinase expression in cellular sensitivity against cisplatin. International Journal of Oncology.

[50] Baek S.M., Kwon C.H., Kim J.H., Woo J.S., Jung J.S., Kim Y.K. (2003). Differential roles of hydrogen peroxide and hydroxyl radical in cisplatin-induced cell death in renal proximal tubular epithelial cells. Journal of Laboratory and Clinical Medicine.

[51] Kilic U., Kilic E., Tuzcu Z., Tuzcu M., Ozercan I.H., Yilmaz O. (2013). Melatonin suppresses cisplatin-induced nephrotoxicity via activation of Nrf-2/HO-1 pathway. Nutrition & Metabolism.

[52] So H., Kim H., Kim Y., Kim E., Pae H.O., Chung H.T. (2008). Evidence that cisplatin-induced auditory damage is attenuated by downregulation of pro-inflammatory cytokines via Nrf2/HO-1. Journal of the Association for Research in Otolaryngology.

[53] Ploemen J.H., van Ommen B., de Haan A., Schefferlie J.G., van Bladeren P.J. (1993). In vitro and in vivo reversible and irreversible inhibition of rat glutathione S-transferase isoenzymes by caffeic acid and its 2-S-glutathionyl conjugate. Food and Chemical Toxicology.

